# Descriptive Analysis of Circulating Antimicrobial Resistance Genes in Vancomycin-Resistant Enterococcus (VRE) during the COVID-19 Pandemic

**DOI:** 10.3390/biomedicines10051122

**Published:** 2022-05-12

**Authors:** Dan Alexandru Toc, Anca Livia Butiuc-Keul, Dumitrana Iordache, Alexandru Botan, Razvan Marian Mihaila, Carmen Anca Costache, Ioana Alina Colosi, Claudia Chiorean, Dan Stefan Neagoe, Liana Gheorghiu, Lia Monica Junie

**Affiliations:** 1 Department of Microbiology, Iuliu Hatieganu University of Medicine and Pharmacy, 400012 Cluj-Napoca, Romania; toc.dan.alexandru@elearn.umfcluj.ro (D.A.T.); botan.alexandru@elearn.umfcluj.ro (A.B.); carmen_costache@elearn.umfcluj.ro (C.A.C.); icolosi@elearn.umfcluj.ro (I.A.C.); mjunie@elearn.umfcluj.ro (L.M.J.); 2 Cluj County Emergency Hospital, 400000 Cluj-Napoca, Romania; mihaila.razvan.marian@elearn.umfcluj.ro (R.M.M.); claudiachiorean@gmail.com (C.C.); danstneagoe@yahoo.com (D.S.N.); gliana66@yahoo.com (L.G.); 3 Department of Molecular Biology and Biotechnology, Faculty of Biology and Geology, Babeş-Bolyai University, 1 M. Kogalniceanu Street, 400084 Cluj-Napoca, Romania; dumitrana.iordache@ubbcluj.ro; 4 Centre for Systems Biology, Biodiversity and Bioresources, Babes-Bolyai University, 5–7 Clinicilor Street, 400006 Cluj-Napoca, Romania

**Keywords:** VRE, *Enterococcus*, resistance genes, *vanA*, *vanB*, COVID-19, Romania

## Abstract

COVID-19 offers ideal premises for bacteria to develop antimicrobial resistance. In this study, we evaluated the presence of several antimicrobial resistance genes (ARG) in vancomycin-resistant *Enterococcus* (VRE) isolated from rectal swabs from patients at a hospital in Cluj-Napoca, Romania. Rectal swabs were cultivated on CHROMID^®^ VRE (bioMérieux, Marcy—l’ Étoile, France) and positive isolates were identified using MALDI-TOF Mass Spectrometry (Bruker Daltonics, Bremen, Germany) and further analyzed using the PCR technique for the presence of the following ARGs: *van A*, *van B*, *tet(M)*, *tet(L)*, *ermB*, *msrA*, *mefA*, *aac(6′)-Im*, *aph(2)-Ib*, *ant(4′)-Ia*, *sul1*, *sul2*, *sul3*, and *NDM1*. We isolated and identified 68 isolates of *Enterococcus faecium* and 11 isolates of *Enterococcus faecalis*. The molecular analysis showed 66 isolates positive for the *vanA* gene and eight positive for *vanB*. The most frequent association of ARG in VRE was *vanA-tet(M)-ermB*. There was no statistically significant difference between *Enterococcus faecium* and *Enterococcus faecalis* regarding ARGs. Our work proves that during the COVID-19 pandemic, highly resistant isolates of *Enterococcus* were present in patients in the intensive care unit; thus, better healthcare policies should be implemented for the management and control of these highly resistant isolates in the future.

## 1. Introduction

*Enterococcus* is genus of Gram-positive cocci, commensals of the human gastrointestinal tract. However, *Enterococci* can produce life-threatening opportunistic infections, particularly nosocomial infections, due to their ability to acquire antimicrobial resistance genes, notably *vanA* for resistance to glycopeptides. Over the past decades, interest in the *Enterococcus* genus has spiked, with the most relevant species, *Enterococcus faecium* and *Enterococcus faecalis*, being in the spotlight due to their antimicrobial resistance and high adaptability features. Other species of the *Enterococcus* genus that harbor an intrinsic resistance to glycopeptides, such as *Enterococcus gallinarum* and *Enterococcus caseliflavus*, and even other rarer findings such as *Enterococcus avium*, *Enterococcus durans*, *Enterococcus raffinosus*, and *Enterococcus cecorum* have been approached in several studies to examine their involvement in human infections [1,2,3].

Since its discovery, vancomycin-resistant *Enterococcus faecium* (VREfm) has been considered as a threat to human healthcare. The vancomycin resistance phenotype is induced by several van operons, with *vanA* and *vanB* being the most common [4]. These operons are involved in the alteration of the peptidoglycan, changing the final amino acid sequence with either D-Alanine-D-Lactate (D-Ala-D-Lac) or D-Alanine-D-Serine (D-Ala-D-Ser) and thus altering the affinity of the glycopeptides, followed by the alteration of the peptidoglycan synthesis. Although in the past, the *vanA* gene was isolated almost everywhere worldwide, with the *vanB* gene being frequent only in some regions such as Australia, recent studies indicate a change in this pattern and thus the need for extensive research regarding these genes worldwide for a better understanding of their distribution and clinical impact [5,6,7,8].

*E. faecium* is intrinsically resistant to a large variety of antibiotics, including aminoglycosides and some beta-lactam antibiotics such as cephalosporins. Since vancomycin was used as a last-resort antibiotic in infections with *E. faecium* for a long time, acquiring vancomycin resistance genes narrows the treatment options to only a few alternatives such as linezolid, tigecycline, or quinupristin/dalfopristin [2]. Recently, there were several isolates of *E. faecium* reported as resistant even to linezolid. These findings show the high risk VREfm poses to humans from treatment and public health perspectives [9,10,11].

Information regarding the circulating antimicrobial resistance genes in Eastern Europe is scarce, and to promote better surveillance policies for VRE, knowing the existing resistance patterns is essential [8,12]. With this study, we aim to provide information regarding the circulating antimicrobial resistance genes in VRE isolates from hospitalized patients during the fourth wave of COVID-19 in Cluj-Napoca, Romania. Due to the extraordinary impact of the COVID-19 pandemic, we are facing a resurgence of multi-drug-resistant (MDR) bacteria, and we must be prepared to tackle this issue appropriately [13].

## 2. Materials and Methods

Rectal swabs of patients admitted to Cluj County Emergency Hospital in the intensive care unit between 1 January 2021 and 1 July 2021, during the COVID-19 pandemic, were cultivated on CHROMID^®^ VRE (bioMérieux, Marcy—l’ Étoile, France). Bacterial isolates were obtained from hospital screening samples through routine clinical protocol and patients’ identifiable information was already anonymized. The isolates were further identified with MALDI-TOF Mass Spectrometry (Bruker Daltonics, Bremen, Germany).

To assess the presence of the antimicrobial resistance genes (ARGs), we used a direct PCR technique. Bacterial isolates were incubated overnight on sheep blood agar (bioMérieux, Marcy—l’ Étoile, France) and several colonies were then suspended in 100 mL of sterile water, adjusted to a 0.5 McFarland concentration. DNA isolation was skipped, and bacterial suspension was used as a template in PCR amplification [14,15]. The PCR reaction mix contained 12.5 μL DreamTaq Green PCR master mix (2x) (Thermo Fisher Scientific, Waltham, MA, USA), 10.25 μL nuclease-free water (Lonza, Basel, Switzerland), 25 pmol of each primer (Eurogentec, Liege, Belgium), and 2 μL bacterial suspension, for a total volume of 25 μL. The PCR reactions were performed in the thermocycler Mastercycler Nexus (Eppendorf AG, Hamburg, Germany). The combinations of primers are displayed in Table 1 and the reaction conditions are shown in Table 2. As a negative control, we used 2 µL of sterile water added to the PCR mixture, and as a positive control, we used 2 µL of the suspension with *vanA*-positive *Enterococcus faecium* ATCC 700221. The PCR amplification was repeated twice.

The amplicons were separated on 1.5% agarose (Cleaver Scientific, Warwickshire, UK) gel in 1× TBE buffer (Lonza, Basel, Switzerland) and stained with 0.5 μg/mL ethidium bromide (Thermo Fischer Scientific, Waltham, MA, USA). The BDA Digital Compact System and BioDocAnalyze Software (Analytik Jena, Germany) were used for data acquisition. Data analysis was performed with IBM SPSS Statistics 26.0 using statistical tests, chi-square or Fisher’s exact test, where appropriate.

## 3. Results

We included in this study 79 isolates of *Enterococcus* that were positive on CHROMID^®^ VRE (bioMérieux, Marcy—l’ Étoile, France), which were further identified as *Enterococcus faecalis* (11 isolates) and *Enterococcus faecium* (68 isolates).

**Figure 1:**
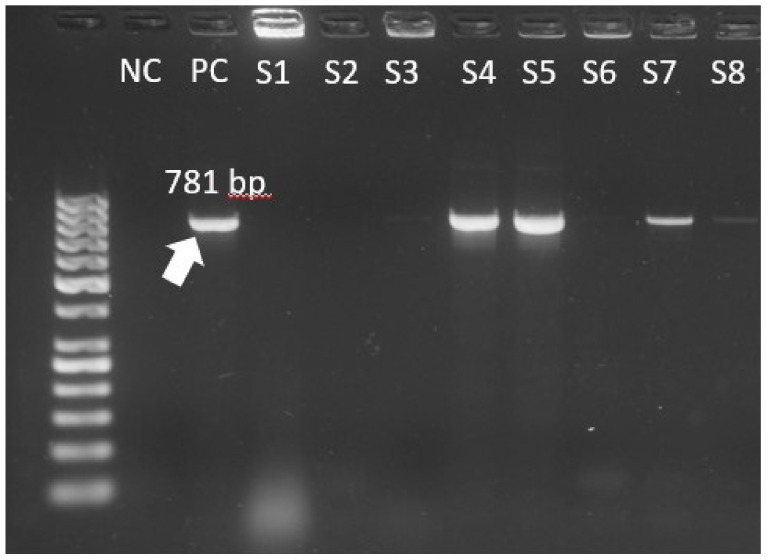
The *vanA* electrophoresis results of *E. faecium* isolates (NC = negative control, PC = positive control, S = sample, bp = base pairs).

**Figure 2:**
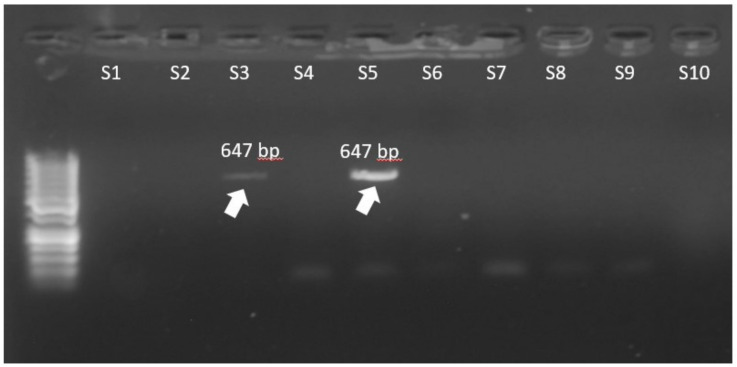
The *vanB* electrophoresis results of *E. faecium* isolates (S = sample, bp = base pairs).

Regarding the resistance to glycopeptides, most of the isolates showed the *vanA* gene (81.8% of *E. faecalis* and 83.8% of *E. faecium*). The *vanB* gene was found only in *E. faecium* (11.8%). Other ARGs encoding resistance to different antibiotics were also studied, as shown in Table 3. The most frequent ARG was *ermB*, present in 97.1% of *E. faecium* isolates and in 81.8% of *E. faecalis* isolates. The electropherograms of *vanA* and *vanB* genes are presented in Figure 1 and Figure 2.

*E. faecalis* isolates showed different patterns of resistance to different classes of antibiotics (Table 4). The most frequent pattern was *vanA-ermB* (27.3%) encoding resistance to vancomycin and macrolides; *vanA-tetM-ermB* (27.3%) encoding resistance to vancomycin, tetracycline, and macrolides; followed by *vanA-ermB-ant4la* (18.2%) encoding resistance to vancomycin, macrolides, and aminoglycosides. The other associations of antimicrobial resistance genes found in *E. faecalis* are presented in Table 4.

The most common associations of ARGs in *E. faecium* were *vanA-tetM-ermB* (29.4%), *vanA-ermB* (17.6%), and *vanA-ermB-ant4la* (14.7%). Other associations are listed in Table 5.

**Figure 3:**
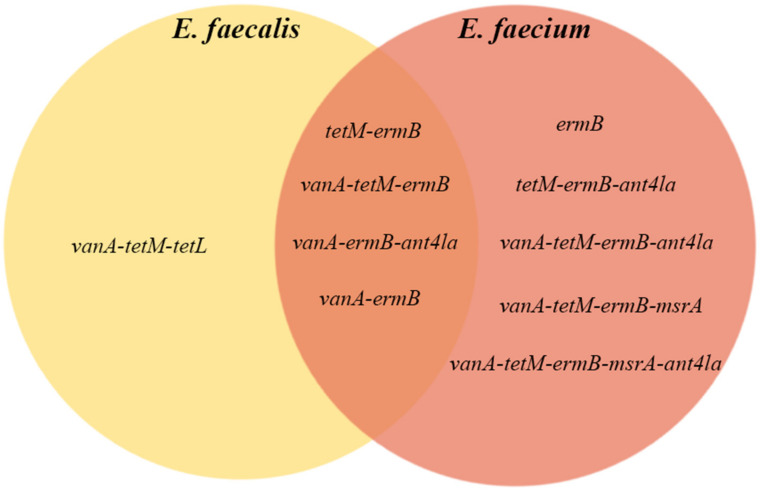
Associations of antimicrobial resistance genes in *E. faecium* and *E. faecalis*.

The ARG associations identified in this study are presented briefly in Figure 3, which shows that the most common associations of ARGs identified for each species also represent three out of the four gene associations identified for both *E. faecalis* and *E. faecium*.

## 4. Discussion

In the past few years, there has been a global effort to reduce the impact of antimicrobial resistance. However, since the beginning of the COVID-19 pandemic, the focus has shifted to ways to decrease the complications and mortality of hospitalized patients that burden medical systems worldwide [16,17,18]. Unfortunately, due to the extended duration of the pandemic, several threats are emerging for human health. One of the most important is the risk of acquisition of new antimicrobial resistance genes. There are several reasons why this risk is certain, and Ukuhor et al. summarize some of the most relevant, including the increased use of antibiotics associated with uncontrolled access to them, mainly in developing countries [19]. Moreover, based on the lessons provided by past pandemics, coinfections with different bacteria or fungi are expected. Moreover, several reports underline the impact of associated bacterial and fungal infections in past pandemics, and similar effects are thus expected now [20,21]. The mortality and morbidity in these situations are extremely high if the microorganisms are resistant to several antimicrobial drugs. One bacterium constantly reported as an associated agent is *Enterococcus faecium* [22]. Rawson et al. underline the importance of a comprehensive analysis of the circulating antimicrobial resistance genes in countries facing the COVID-19 pandemic to install proper guidelines designed for those regions in particular [17].

Vancomycin is a glycopeptide used to treat infections produced mostly by Gram-positive bacteria. Its mechanism of action relies on binding to the terminal sequence D-Ala-D-Ala of the peptidoglycan, therefore inhibiting the bacterial cell wall synthesis [14]. Resistance to vancomycin was reported in enterococci in the late 1980s and since then, it has become a serious public health issue due to the life-threatening infections produced by vancomycin-resistant enterococci. Resistance to vancomycin involves an alteration in the terminal sequence of the peptidoglycan, changing the structure from D-Ala-D-Ala to D-Ala-D-Lac (for *vanA*, *vanB*, *vanD*, and *vanM*) or D-Ala-D-Ser (for *vanC*, *vanE*, *vanG*, *vanL*, and *vanN*). This mutation lowers the affinity of vancomycin to the binding site and thus promotes a resistant phenotype [8,23,24].

Our study analyzed the *vanA* and *vanB* genes in *E. faecalis* and *E. faecium*. For *E. faecalis*, *vanA* was the only one present, being positive in 9 out of 11 isolates. Similarly, *vanA* was dominant in *E. faecium*, being present in 57 out of 68 isolates. We also found eight *E. faecium* isolates that were positive for the *vanB* gene. These results are similar to the existing literature showing that in Romania, circulating isolates of VREfm predominantly harbor the *vanA* gene [12,25]. Statistically, no significant difference was determined between *E. faecalis* and *E. faecium* regarding the *vanA* and *vanB* genes.

Interestingly, two of these isolates were positive for both *vanA* and *vanB* genes. This observation was previously described in rare occasions with high clinical significance [26,27]. Unfortunately, we did not determine the phenotype expressed by this combination. Still, this observation implies high genome mobility between the species in the hospital environment [28,29,30].

Another relevant observation is that there are two isolates of *E. faecalis* and five isolates of *E. faecium* that were negative for both *vanA* and *vanB* genes. Based on the existing literature, we hypothesize that this finding might be due to the existence of other vancomycin resistance genes that we did not test for [24].

Regarding the resistance to tetracyclines, two mechanisms were tested in this study: drug efflux mediated via the *tet(L)* gene and target protection mediated via the *tet(M)* gene [8,31]. In *E. faecalis* isolates, *tet(M)* was the most frequent, being present in 5/11 isolates, while *tet(L)* was present only in one strain. Similarly, *E. faecium* isolates were positive for *tet(M)* in 38/68 isolates, while *tet(L)* was present in only 5/68 isolates. Although we can conclude that *tet(M)* is the most frequent resistance gene in this population of *Enterococci*, we did not observe any statistically significant difference between *E. faecium* and *E. faecalis* regarding these two genes (*p* > 0.05).

It was previously shown by Molechan et al. that *tet(M)* is often associated with *ermB* [32]. In our study, this association was present in four isolates of *E. faecalis* (three associated also with the *vanA* gene) and 35 isolates of *E. faecium.* This association has clinical relevance, with these isolates being resistant to a wider variety of antimicrobial agents. However, due to the lack of multilocus sequence typing (MLS) or whole-genome sequencing (WGS), we are not able to provide more extensive molecular epidemiology information concerning these isolates.

Tigecycline is a new antibiotic from the class of tetracyclines, which is effective against *tet(M)*- or *tet(L)*-positive isolates due to the low affinity for the efflux proteins and the ribosomal protection mechanisms [24,33]. Thus, it is used in different types of infections with *Enterococcus*, such as endocarditis and intra-abdominal infections [34]. Recently, Fiedler et al. demonstrated that some isolates harboring both *tet(M)* and *tet(L)* genes are resistant to tigecycline [35]. Our study shows one isolate of *E. faecalis* and five isolates of *E. faecium* that display this genotype, being positive for both *tet(M)* and *tet(L)*. The presence of these associations in strains isolated from hospitals shows the importance of molecular analysis of antimicrobial resistance genes to implement the best long-term healthcare policies.

Resistance to macrolide–lincosamide–streptogramin antibiotics was evaluated by the presence of *mefA*, *msrA*, and *ermB* genes. While *mefA* and *msrA* genes are responsible for producing an active efflux pump mechanism, the *ermB* gene produces a methylase that modifies the drug’s target [36,37,38,39]. In our study, only 9 out of 11 *E. faecalis* isolates were positive for *ermB*. We also noticed the association of *vanA* and *ermB* genes in seven of those nine isolates. This pairing was also described by Dziri et al. and Lopez et al. [40,41]. We hypothesize that these associations, most described in *E. faecium*, were possible due to the horizontal gene transfer. A total of 66 out of 68 isolates of *E. faecium* presented the *ermB* gene, being the most prevalent ARG out of all that we tested. Moreover, the *vanA*-*tetM*-*ermB* association was present in 20 isolates, and an additional 13 isolates presented the same association in combination with other genes. These findings are in accordance with other studies and usually describe highly resistant isolates of *E. faecium* [24,40,41]. In our study, there was no statistically significant difference between *E. faecalis* and *E. faecium* concerning the *mefA*, *msrA*, and *ermB* genes.

Enterococci are intrinsically resistant to aminoglycosides due to the poor penetration through the bacterial cell wall. However, aminoglycosides proved to be useful in the clinical setting in combination with active agents against the cell wall. Thus, resistance to aminoglycosides is the consequence of modifying enzymes that work against this synergistic benefit. In our study, we evaluated the presence of three genes related to the aminoglycoside resistance, namely, *aac(6′)-Im*, *aph(2)-Ib*, and *ant(4′)-Ia*. The first two genes are responsible for the synthesis of an acetyltransferase and a phosphotransferase, respectively, and they provide resistance to most aminoglycosides (with the notable exception of streptomycin). The *ant(4′)-Ia* gene encodes a nucleotidyltransferase responsible for resistance to streptomycin [42,43,44,45,46,47]. In our study, none of the isolates tested presented the *aac(6′)-Im* or *aph(2)-Ib* genes. On the other hand, *ant(4′)-Ia* was positive in 2 out of the 11 *E. faecalis* isolates and in 20 out of 68 isolates of *E. faecium*. The association of *vanA-ermB-ant(4′)-Ia* was the most frequent among the isolates positive for aminoglycoside resistance, being present in both isolates of *E. faecalis* and in 10 out of the 20 isolates of *E. faecium*. This genotype is often translated in a phenotype resistant to glycopeptides, macrolides, and aminoglycosides, being difficult to treat in a clinical setting. No statistically significant difference between *E. faecalis* and *E. faecium* regarding the *ant(4′)-Ia* gene was found in our study.

Usually, antimicrobial resistance genes are transferred horizontally through different mobile genetic elements. More often, *vanA* operon is associated with a transposon (Tn), Tn1546 [33]. For *vanB*, there are different Tns that facilitate the transfer of the gene, such as Tn1547 and Tn1549 [8]. There are other possible gene associations that may use transposons as means of dissemination, such as *tet(M)* and *ermB*, as previously stated [32]. All these observations suggest a frequent horizontal transfer present in the intensive care unit of our hospital that needs to be monitored and addressed according to the existing guidelines.

Intensive care units represent an environment of intense antibiotic pressure, which provides ideal premises for acquiring different antimicrobial resistance genes via horizontal or vertical transfer, described by Rehman et al. as the main routes of dissemination of resistance [48]. For this reason, we also checked the presence of the sulfonamide resistance genes (*sul1*, *sul2*, and *sul3*) and carbapenem resistance via the New Delhi metallo-beta-lactamase gene (*NDM-1*) in vancomycin-resistant enterococci, despite their natural resistance to these antibiotic classes. *sul1, sul2,* and *sul3* are the only known genes that provide resistance to sulfonamides by producing an alternative dihydropteroatesynthase [49,50,51,52]. The *NDM-1* gene produces an enzyme capable of hydrolyzing the carbapenem antibiotics [53]. It was first reported by Yong et al. in 2009 [54] and it quickly became a major public health issue. Although it is usually harbored by Gram-negative Enterobacteriaceae, Walsh et al. describe other possible environmental hosts for this gene, such as *Kingella dentrificans* or *Sutonella indologenes* [55]. Even if highly unlikely, the presence of these genes in Gram-positive bacteria has the potential to pose a new challenge in fighting antimicrobial resistance. In our study, none of the isolated strains presented the *sul1*, *sul2*, *sul3*, or *NDM1* gene.

Genotypic analyses of *Enterococcus* strains isolated from Romania are scarce. The few articles that tackle the antimicrobial resistance profile of *Enterococcus* species usually approach only the phenotype, and based on them, the resistance to glycopeptides seems to be low [56,57,58,59]. However, regarding the genotype of the resistance to glycopeptides in Romania, Ducu et al. found that the *vanA* gene was present in 19 out of 84 *Enterococcus* isolates and the *vanB* gene was present in 10 out of 84 *Enterococcus* isolates [25]. Based on that, we observe that before COVID-19, resistance to glycopeptides was relatively low, in opposition to our study, which shows a higher presence of *vanA* and *vanB* genes alone and in association with other ARGs in screening samples from ICU patients during the COVID-19 pandemic. This suggests that in the post-COVID-19 era, we might experience infections with highly resistant isolates of *Enterococcus*.

Regarding the limitations of our study, the most relevant is the relatively small number of isolates collected in a short period from one facility only. Moreover, the lack of multilocus sequence typing or whole-genome sequencing prevents a better understanding of the epidemiology of these isolates.

On the other hand, the strengths of the study include the number of ARGs tested and the efficient method of identification of the isolates, thus providing accurate and comprehensive information regarding the circulating ARGs in VRE during the COVID-19 pandemic in Cluj-Napoca, Romania.

## 5. Conclusions

*Enterococcus faecalis* and *Enterococcus faecium* are the main species of the genus *Enterococcus,* considered the most important in terms of antimicrobial resistance and potential healthcare threats. The COVID-19 pandemic offers an unprecedented ground for developing highly resistant isolates considering the irresponsible use of antibiotics and the pressure on the healthcare system.

Based on our analysis, in the first six months of 2021, during the COVID-19 pandemic in Romania, the dominant species isolated from rectal screening was *Enterococcus faecium*. These isolates harbored mostly the *vanA* gene, and the most prevalent association of antimicrobial resistance genes was *vanA-tet(M)-ermB*. Although *Enterococcus faecalis* was isolated less frequently, the clinical importance remains due to the high prevalence of the *vanA* gene. Based on our analysis, the most frequently associated antimicrobial resistance genes in vancomycin-resistant *Enterococcus faecium* and *Enterococcus faecalis* are, in order, *ermB*, *tet(M)*, and *ant(4′)-Ia*.

To our knowledge, this paper provides the most extensive analysis of circulating antimicrobial resistance genes in *Enterococcus* in Romania. According to our results, the COVID-19 pandemic seems to produce a dangerous aftermath in terms of highly resistant strains of *Enterococcus* that may be a threat in the near future.

## Figures and Tables

**Table 1:** Characteristics of the primers used for the antimicrobial resistance genes’ amplification and the dimension of the amplicons (bp—base pairs).

Gene	Primer Pairs		Amplicon (bp)
	Forward	Reverse	
*vanA*	GCTATTCAGCTGTACT	CAGCGGCCATCATACGG	781
*vanB*	CGCCATATTCTCCCCGGATAG	AAGCCCTCTGCATCCAAGCAC	647
*tet(M)*	CCGTCTGAACTTTGCGGAAA	CAACGGAAGCGGTGATACAG	627
*tet(L)*	TATTCAAGGGGCTGGTGCAG	CGGCAGTACTTAGCTGGTGA	545
*ermB*	GAAAAGGTACTCAACCAAATA	AGTAACGGTACTTAAATTGTTTAC	639
*msrA*	AGGGAAAGGTCATTTTACTGC	CCCTACCTATAACTAAACATT	343
*mefA*	CATCGACGTATTGGGTGCTG	CCGAAAGCCCCATTATTGCA	516
*aac(6′)-Im*	GGCTGACAGATGACCGTGTTCTTG	GTAGATATTGGCATACTACTCTGC	482
*aph(2)-Ib*	CTGAACACAGCAGCGACTAC	TTGTAATCGCCATGCACCAG	646
*ant(4′)-Ia*	GTCAAAAACTGCTAACACAAG	AATAATACTGCTAACGATAAT	135
*sul1*	AGGCATGATCTAACCCTCGG	GGCCGATGAGATCAGACGTA	665
*sul2*	GACAGTTATCAACCCGCGAC	GAAACAGACAGAAGCACCGG	380
*sul3*	GTGGGCGTTGTGGAAGAAAT	AAAAGAAGCCCATACCCGGA	370
*NDM-1*	GGTTTGGCGATCTGGTTTTC	CGGAATGGCTCATCACGATC	621

**Table 2:** PCR conditions used for each antimicrobial resistance gene tested.

Gene	Initial Denaturation	Steps (30 Cycles)	Final Elongation
*vanA*, *ermB*	94 °C for 4 min	Denaturation at 94 °C for 1 min Annealing at 51 °C for 45 s Elongation at 72 °C for 45 s	72 °C for 8 min
*ant(4′)-la*, *msrA*		Denaturation at 94 °C for 1 min Annealing at 53 °C for 45 s Elongation at 72 °C for 45 s	
*aph(2)-lb*, *mefA*, *TetM*, *sul3*		Denaturation at 94 °C for 1 min Annealing at 55 °C for 45 s Elongation at 72 °C for 45 s	
*TetL*, *sul1*, *sul2*		Denaturation at 94 °C for 1 min Annealing at 57 °C for 45 s Elongation at 72 °C for 45 s	
*aac(6′)–Im*, *vanB*		Denaturation at 94 °C for 1 min Annealing at 61 °C for 45 s Elongation at 72 °C for 45 s	

**Table 3:** Number and percentage of *E. faecalis* and *E. faecium* isolates positive for the ARGs among the total number of isolates tested.

	*Enterococcus Faecalis* (*n* = 11)	*Enterococcus Faecium* (*n* = 68)	*p*
*van A*	9 (81.8%)	57 (83.8%)	1.000
*van B*	0 (0%)	8 (11.8%)	0.591
*tet(M)*	5 (45.5%)	38 (55.9%)	0.529
*tet(L)*	1 (9.1%)	5 (7.4%)	1.000
*ermB*	9 (81.8%)	66 (97.1%)	0.091
*msrA*	0 (0%)	2 (2.9%)	1.000
*mefA*	0 (0%)	1 (1.5%)	1.000
*aac(6′)-Im*	/	/	N/A
*aph(2)-Ib*	/	/	N/A
*ant(4′)-Ia*	2 (18.2%)	20 (29.41%)	0.718
*sul1*	/	/	N/A
*sul2*	/	/	N/A
*sul3*	/	/	N/A
*NDM1*	/	/	N/A

**Table 4:** Number of *E. faecalis* isolates and percentage from all the included isolates for each ARG combination.

	Frequency	Percentage
Resistance gene	1	9.1%
*tetM-ermB*	1	9.1%
*vanA-tetM-tetL*	1	9.1%
*vanA-ermB-ant4la*	2	18.2%
*vanA-ermB*	3	27.3%
*vanA-tetM-ermB*	3	27.3%
Total	11	100.0%

**Table 5:** Number of *E. faecium* isolates and percentage from all the included isolates for each ARG combination.

	Frequency	Percentage
Resistance gene	1	1.5%
*tetM-ermB*	1	1.5%
*tetM-ermB-ant4la*	1	1.5%
*vanA-tetM-ermB-msrA*	1	1.5%
*vanA-tetM-ermB-msrA-ant4la*	1	1.5%
*vanA-tetM-tetL-ermB-ant4la*	1	1.5%
*vanA-tetM-tetL-ermB-mefA*	1	1.5%
*vanA-vanB-ermB*	1	1.5%
*vanA-vanB-tetM-ant4la*	1	1.5%
*ermB*	2	2.9%
*vanB-tetM-ermB*	2	2.9%
*vanA-tetM-tetL-ermB*	3	4.4%
*vanB-ermB*	4	5.9%
*vanA-tetM-ermB-ant4la*	6	8.8%
*vanA-ermB-ant4la*	10	14.7%
*vanA-ermB*	12	17.6%
*vanA-tetM-ermB*	20	29.4%
Total	68	100.0%

## Data Availability

Not applicable.
